# The Genetics of *PPARG* and the Skeleton

**DOI:** 10.1155/PPAR/2006/93258

**Published:** 2006-10-11

**Authors:** Cheryl Ackert-Bicknell, Clifford Rosen

**Affiliations:** ^1^The Jackson Laboratory, Bar Harbor ME 04609, USA; ^2^St. Joseph's Hospital, The Maine Center for Osteoporosis Research and Education, Bangor ME 04401, USA

## Abstract

Osteoporosis is a complex metabolic bone disorder. Recently it has been appreciated that the “obesity in bone” phenomenon occurs at the expense of bone formation, and that is a key component of the pathology of this disease. Mouse models with altered bone expression levels of peroxisome proliferator-activated receptor gamma (PPARG) impact bone formation, but genetic studies connecting *PPARG* polymorphisms to skeletal phenotypes in humans have proven to be less than satisfactory. One missense polymorphism in exon one has been linked to low bone mineral density (BMD), but the most studied polymorphism, Pro12Ala, has not yet been examined in the context of skeletal phenotype. The studies to date are a promising start in leading to our understanding of the genetic contribution of *PPARG* to the phenotypes of BMD and fracture risk.

## INTRODUCTION

Osteoporosis currently affects 10 million Americans and an additional 34 million Americans are considered at risk for osteoporosis and fracture (http://www.nof.org/ accessed June, 2006). The World Health Organization (WHO) defines osteoporosis as having a BMD with a T-score of less than −2.5 [1], yet in the Rotterdam prospective study of 7806 men and women over the age of 55, only 44% of women and 21% of the men with a nonvertebral fracture had a T-score of −2.5 or lower [2] suggesting a need for additional means for predicting fracture risk. A variety of studies have been done to examine other risk factors for osteoporosis, both for the purpose of determining who should undergo further screening and more importantly, who is at risk for fracture. Osteoporosis and the clinically measurable phenotypes such as BMD and fracture incidence have proven to be very complicated genetic traits with quantitative trait loci (QTLs) for various bone phenotypes found on almost every chromosome in both humans and mice (reviewed in [3, 4]). Yet BMD is not an independent phenotype, rather it is associated with many other phenotypes and pathologies such as diabetes mellitus [5] and coronary artery disease [6]. Body weight is positively correlated to bone mass and in load-bearing skeletal sites, increased adiposity is associated with higher BMD, yet adiposity still influences BMD at non-load-bearing sites such as the forearm [7]. PPARG's role in insulin sensitivity and obesity, as well as work done with mesenchymal stem cells have made PPARG an attractive candidate gene in studies examining the genetic basis of bone density.

Meunier et al [8], were the first to show that women with osteoporosis had an increased accumulation of marrow adipocytes as determined from iliac crest biopsies [8]. More recent studies have not only confirmed this observation, but have also shown that volume fraction of the marrow cavity occupied by adipocytes increased with age in both men and women and that this is coincident with a decrease in trabecular bone volume. This increase in adipocyte volume is exacerbated in osteoporotic patients [9, 10]. More importantly, the increased adipocyte volume seen in osteoporotic patients is negatively correlated with bone formation rate (BFR) [10].

Osteoblasts, the cells responsible for the formation of bone, are derived from marrow mesenchymal stem cells. This multipotential stem cell is also able to give rise to chondrocytes, muscle cells, marrow stromal cells, and adipocytes [11]. Lineage allocation is determined by the activation of lineage-specific transcription factors such as RUNX2 (CBFA1), an osteoblast-specific transcription factor or PPARG, a nuclear receptor shown to be key for the maturation of adipocytes [12, 13]. In preosteoblast cell lines, it has been shown that expression of PPARG2 can force a commitment to the adipogenic pathway [14], an occurrence that can be mimicked by the addition of the pharmacological PPARG ligand BRL4965 [15]. In studies of aging mice, it has been shown that the increase in adipocyte volume in the bone marrow seen with aging is coincident with an increase in expression of PPARG2 [16].

## *PPARG* GENE STRUCTURE, FUNCTION, AND GENETIC LOCATION

PPARG is one of three PPAR nuclear receptors and while widely expressed, it is primarily found in white adipose tissue. Like all nuclear receptors, PPARG is composed of three domains: the N-terminal domain A/B domain, a two-zinc finger containing DNA-binding domain, and a C-terminal ligand-binding domain (17–19). PPARG forms a heterodimer with the retinoic X receptor-alpha and this complex binds to the PPRE (PPAR response element), a direct repeat of the sequence AGGTCA separated by a single nucleotide spacer, in the target gene [17]. Several classes of compounds, both endogenous and exogenous, have been found to act, at least in part, as ligands for PPARG and included polyunsaturated fatty acids such as arachidonic acid, prostaglandins-like compounds, oxidized lipids such as 9-HODE, and the widely used pharmacological thiazolidinedione (TZD) compounds (20).

**Figure 1:**
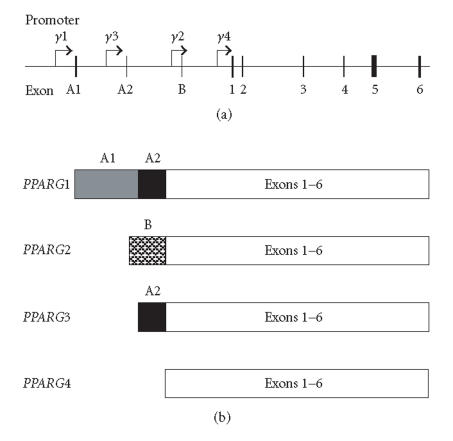
A schematic representation of the *PPARG* gene. (a) The *PPARG* gene is composed of nine exons, named A1, A2, B, 1, 2, 3, 4, 5, and 6, respectively, and four promoters. (b) There are four major *PPARG* transcripts, all of which contain exons 1 through 6. Expression of each transcript is controlled by one of the four promoters. All of the transcripts yield the same protein, except for the γ2 transcript, which codes for 30 additional amino acids on the N-terminus.

*PPARG* is located in humans on 3p25.3 at Mb position 12.3 to 12.45 and in mouse on chromosome 6 at 115.8 to 115.93 Mb (http://www.ensembl.org v.37, release date: February, 2006). The gene is composed of nine exons, four promoters and yields four transcripts via alternate promoter use and splicing [18–20]. All transcripts contain the exons numbered one through six. It is the alternate promoters and leader exons that yield the four distinct transcripts. As shown in Figure 1, *PPARG*1 is transcribed from the g1 promoter and consists of exons A1, A2 and the ubiquitous exons one through six [18, 19] and is considered to be universally expressed [20]. *PPARG*2, which is only found in adipose tissue [21], is transcribed from the third promoter, which is referred to as g2, and consists of exon B and exons one through six [18, 19]. *PPARG*3, also ubiquitously expressed [20], is transcribed from the second promoter g3 and consists of exons A2 and one through six [19]. The last isoform characterized in humans *PPARG*4 does not contain any of the three leader exons, and rather is expressed directly from the g4 promoter found immediately in front of exon one [20]. Little is known about the g4 transcript, although a recently characterized mutation in humans suggests a key role for this transcript in adipocyte biology [22]. All of the transcripts of *PPARG*, with the exception of the transcript generated from the g2 promoter, yield the same protein product. The protein product yielded by the g2 promoter's transcript PPARG2 contains 30 extra amino acids on the N-terminus. These extra 30 amino acids have been shown to increase the transcriptional activity of PPARG2 by 5–10-fold over that of PPARG1 (26).

## GENETIC MAPPING STUDIES IN HUMANS

Of all of the many genome wide scans published to date, only Deng et al [23] report a QTL for BMD in the vicinity of the *PPARG* gene. They showed a forearm-specific BMD QTL with a peak at D3S1297 (3p26) with a modest LOD score of 1.82 [23]. A recent meta analysis was done by Lee et al using data from 11 separate genome-wide scan studies comprised of 3097 families with 12 685 individuals of a variety of ethnic backgrounds [24]. These investigators found suggestive evidence for a QTL for BMD in human on 3p25.3 to 3p22.2, the exact region of the *PPARG* gene. The study by Deng et al was not one of the studies used in this analysis [24]. While studies have examined the heritability of fracture risk [4], no study to date has mapped a QTL for fracture risk to 3p25.

**Figure 2:**
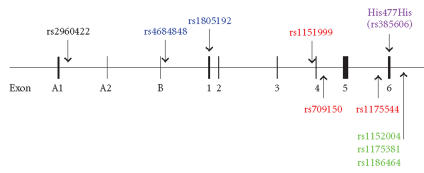
Physical location of the studied human *PPARG* polymorphisms. Several of these SNPs have been shown to be in high LD. All SNPs within an LD block are shown as the same color.

Several mutations have been discovered in *PPARG* in human and have been investigated for their role in obesity, diabetes, and metabolic syndrome and as such are reviewed elsewhere [25]. Four studies published to date have investigated the genetic association of *PPARG* polymorphisms and bone in humans, as summarized in Figure 2 and Table 1.

A silent His477His (C → T, rs3856806) mutation has been identified in humans in the 161st base pair (bp) of the sixth exon of *PPARG* and is referred to in the literature as C161T (as numbered from the beginning of exon 6) or C1431T (as numbered from the ATG start site). While this single nucleotide polymorphism (SNP) may actually be in linkage disequilibrium (LD) with another more causative mutation, the T allele has been associated with increased plasma leptin and adipose tissue mass [30] as well as improved lipid profiles in type II diabetes [31, 32]. Two studies have examined this polymorphism in the context of bone. In the first study of 394 postmenopausal Japanese women, an association between carriers of at least one T allele and increased total body BMD was observed [26]. A more recent study of 138 premenopausal and 125 postmenopausal Korean women showed no association with this SNP and any marker of bone formation, bone resorption, or BMD at the spine or hip, with the exception of serum osteoprotegerin (OPG) [27]. In this study, the authors showed a relationship between low OPG levels and the T allele [27]. While these two studies contradict one another, it must be remembered that first, the cohort size in these studies were very small and second, this is a silent polymorphism and is likely in LD with a more causative mutation. Studies with larger sample sizes and studies involving different ethnic groups must be done in order to get a more comprehensive picture regarding any association of this SNP with bone biology.

Two studies have looked at associations between SNPs in the *PPARG* gene and bone in larger human cohorts. A study of 6743 Chinese men and women examined a single SNP upstream of the first promoter of *PPARG* (rs2960422) and showed a modest increase in the risk of low BMD with the heterozygous state of this allele, but only in premenopausal women. No association was found in either men or postmenopausal women [28]. It must be noted that to date, this SNP has only been examined in this one ethnic group.

A more comprehensive study of SNPs in *PPARG* and their association with aspects of bone density has been done in the Framingham Offspring cohort [29]. The population of study consisted of 740 men and 776 women, with an average age of 61 years old, who were primarily Caucasians. Eight SNPs constituting three LD blocks were investigated for association with femoral neck, greater trochanter or spine BMD as well as with broadband ultrasound attenuation (BUA) of the calcaneus. The location of these SNPs and the LD blocks is summarized in Figure 2. Only one coding SNP was assessed in this study, rs1805192. This SNP, located in the universal exon one, codes for the substitution of an alanine (Ala) for the wild-type proline (Pro) but is not to be confused for the much-studied Pro12Ala polymorphism found in exon B [29]. Homozygosity for the more common Pro allele was associated with increased BMD at both the femoral neck and lumbar spine as well as increased BUA in women, when the data was adjusted for age and estrogen status. Conversely, men with this same allele had lower femoral neck and trochanter BMD [29]. A full examination of this amino acid substitution has not been undertaken to date but computer modeling programs designed to predict the implications of amino acid change suggest that this substitution could have structural consequences [33, 34]. The C allele of the SNP rs1175381 located distal to the polyadenylation signal was associated with lower BMD at all sites measured in women. No association with men was reported [29]. Lastly, a haplotype block of three SNPs with the associated alleles shown in brackets, rs1151999 (A), rs709150 (C) and rs1175544 (C), was in women, also associated with lower BMD of the femoral neck, trochanter, and lumbar spine, but no association was found in men. Interestingly all of these allele-BMD associations were found to be independent of BMI or type II diabetes (36).

All of the findings presented in these four studies need to be confirmed in other cohorts. Both the Chinese cohort study and the Framingham study are ongoing studies and it is hoped that future publications from these two groups will include an examination of such well-studied SNPs such as the Pro12Ala and the His477His SNP. While these studies did correct for factors such as menopausal status, there may well be other confounding and/or interacting factors that have not been taken into account in these studies, thus masking important results. Previous studies have shown *PPARG* allele by environment interactions for a variety of non bone phenotypes, warranting more comprehensive studies of this gene and bone [35–37].

## BONE BIOLOGY OF THE *Pparg* KNOCKOUT ANIMAL

Homozygous knock out *Pparg^tm1Tka^* mice die at embryonic day 10.5 to 11 pc due to placental insufficiency and cardiac defects, making any meaningful examination of skeletal biology impossible [38]. In contrast, the *Pparg* heterozygous knockout mouse (*Pparg* ^+/−^) is viable and appears to have normal development of all major organs. Akune et al have thoroughly examined the bone biology of this haploinsufficient *Pparg* mouse [39]. The *Pparg* ^+/−^ male mice show marked increase in trabecular bone volume at 8 weeks of age as compared to wild-type, and while the volume fraction of trabecular bone (BV/TV) of the distal femur did decrease with age in both genotypes, the *Pparg* ^+/−^ mouse maintained a higher BV/TV than the wild-type controls through 52 weeks of age. Histological analysis showed a more than 50% increase in the number of osteoblasts and a doubling in the total bone formation rate (BFR) of the haploinsufficient mice, leading to the conclusion that the function of individual osteoblasts was not affected. This increase in osteoblast number was coincident with a trend for a decrease in adipocyte number. The number of adipocytes in the marrow increased in the wild-type controls with age, but no change in adipocyte number was observed in the *Pparg* ^+/−^ mice by 52 weeks of age. The effects of estrogen loss in females on bone, in the context of low PPARG were also examined. The loss of one *Pparg* allele was not protective to bone, as the *Pparg* ^+/−^ ovariectomized (OVX) mice lost the same proportion of bone after OVX, as the wild-type OVX mice lost when compared to the appropriate genotypic sham operated mice [39]. Although Rieusset et al, in a separate study, report slight total body growth retardation in the *Pparg* ^+/−^ male but not female mice [40], Akune et al found no such growth retardation.

## SENESCENCE-ACCELERATED MOUSE P6

The senescence-accelerated series of mice (SAM) were created in the 1970s as model for the study of physiological decline with aging. Two series of mouse lines were created: the SAMR series served as control lines and the SAMP lines were selected for signs of advanced aging. The SAMP6 line was created from the SAMR3 line, from a pedigree that showed spontaneous leg fractures with advanced age [41]. While indistinguishable from the SAMR1 control strain at one month of age, bones from the SAMP6 mice showed decreased trabecular bone volume, decreased cortical thickness, lower areal BMD, and lower BFR as early as three months of age. The SAMP6 mice also showed a decreased bending strength and increased brittleness, and are considered an excellent model of the senile osteoporosis observed in humans [42, 43]. The SAMP6 mice show an increase in marrow adiposity with aging [44] and a coincident decrease in osteoblast precursor cells evident as early as three months of age [42]. More recently, it has been shown that *Pparg2* mRNA levels increase in the marrow with aging in these mice, yet this could be blocked by a yet-to-be-determined mechanism upon the administration of 1, 25(OH)_2_D_3_ (49).

## MAPPING STUDIES IN MICE

Two separate mouse mapping crosses in mice have identified a QTL for an aspect of bone density or geometry on the distal 6th chromosome (Chr) in the vicinity of the *Pparg* gene. Klein et al have identified QTL for femoral cross-sectional area, with a broad peak that includes the genetic location of *Pparg* in a C57BL/6J (B6) by DBA/2J cross [45]. Drake et al have shown a QTL for bone density that colocalized with adipose tissue mass and bone torsional strength QTLs in the same genetic location as Klein et al in a cross of the same two strains, but only after the mice were fed a high fat diet [46].

Our laboratory has conducted intensive studies of a Chr 6 QTL found in a cross of B6 by C3H/HeJ (C3H), *Bmd*8 [47]. A congenic mouse was made for the purpose of studying this QTL in isolation from the large number of other BMD affecting QTLs found on other chromosomes. The ensuing strain B6.C3H-6T (6T) was made by introgressing the region of 6th Chr encompassed by the markers *D6Mit*93 and *D6Mit*150 from C3H onto a B6 background by 9 generations of selective backcrossing, followed by several generations of intercrossing. The resulting mouse is homozygous for B6 alleles for the entire genome except for the region between *D6Mit*93 and *D6Mit*125, which is homozygous for the C3H alleles [48]. The biology of the 6T mouse has been well studied. This strain has lower BMD than either the B6 background strain, or the C3H donor strain. 6T mice have a smaller periosteal circumference, slightly shorter femurs, and a lower BFR as compared to the B6 background strain [48]. There are several candidate genes in the congenic region of the 6T mouse for the various phenotypes seen in the 6T mouse, including, but not limited to *Pparg*, arachidonate 5-lipoxygenase (*Alox5*), adiponectin receptor 2 (*AdipoR2*), and *Wnt5b*. While not all of the phenotypes seen in the 6T mouse can be explained by a single gene alteration, the 6T mouse does have a strikingly opposite phenotype than that seen in the *Pparg* ^+/−^ mouse for several key phenotypes. For example, the 6T mouse has increased numbers of marrow adipocytes and significantly lower trabecular bone volume at all sites measured when compared to the background strain [48, 49]. Marrow stromal cell cultures show that there are less alkaline phosphatase staining colonies as compared to B6 control cultures as soon as 7 days after culture, suggesting a decrease in osteoblastogenesis [49].

Yet the biology of the 6T mouse is not clear cut. Increased fat feeding (increase in % kcal from fat), which provides more exogenous ligand for *Pparg*, does not increase total body fat in the female 6T mouse, as it does in the B6 control strain, nor does it affect the number of marrow adipocytes. However, decreased fat feeding does improve the BV/TV in 6T to levels seen in control fed B6 mice [50]. Differences in *Pparg* transcript levels have been found in both the liver and in the bone when comparing 6T back to the background B6 strain [49]. In addition, several polymorphisms in both coding and noncoding regions of *Pparg* have been found when comparing B6 to C3H. While no nonsynonymous SNPs have been found, several intriguing promoter polymorphisms have been found as well as 12 SNPs in the 3′ UTR (Ackert-Bicknell, unpublished data). Both the biology of the 6T mouse as well as the number of polymorphisms in *Pparg* suggest a key role for this gene in the bone phenotype of the 6T mouse.

Our original F2 genetic mapping-cross suggested that this Chr 6 QTL interacted with a locus on the 11th Chr (56). The *Alox15* gene, which codes for an enzyme key in the formation of 15S-HETE, an endogenous ligand for PPARG (57), is located on Chr 11 at 70069811–70077674 Mb (http://www.ensembl.org v.37, release date: February, 2006) and knockout mice for this gene show higher femoral BMD and femoral stiffness [51]. Associations with BMD have been found in human with SNPs in *ALOX12*, the gene that codes for the human functional homologue to the mouse *Alox15* [52]. Another member of the ALOX gene family, *Alox5*, is located approximately 1 Mb distal to *Pparg* on mouse Chr 6 and also likely produces a ligand for PPARG. While expression of *Alox15* is much more widespread, the expression of *Alox5* appears to be more limited with the greatest expression levels seen in bone and white blood cells (http://symatlas.gnf.org/SymAtlas/).

It is interesting to speculate about the causative gene or genes in the 6T mouse. In some ways, the phenotypes of the 6T mice mimic phenotypes of the *Pparg* ^+/−^ mouse, such as the resistance fat feeding induced obesity [50, 53], yet in other respects, the 6T mouse is the exact opposite of the *Pparg* ^+/−^ mouse. Are alterations in the *Pparg* gene the cause of this, or is PPARG the mediator of this action under the control of another gene, such as a member of the *Alox* gene family? Cellular differentiation in bone cell lineages, as driven by PPARG, has been shown to be dependant on the type of PPARG ligand present [54], further suggesting the alterations in ligand processing and/or the ability of PPARG to respond appropriately, may contribute to the interesting physiology of the 6T mouse. Additional experiments are in progress to elucidate the genetic mechanisms responsible for the phenotypes seen in the 6T mouse.

## PPARG, DIABETES, AND OBESITY

The Pro12Ala polymorphism has been found in a variety of ethnic populations [25] and has been shown to decrease both the binding of PPARG/RXR heterodimers to the PPRE and their ability to activate gene transcription [55]. This polymorphism has not been studied with regard to an association with bone density, but it has been examined in the context of several other physiological and pathological states that are known to impact bone health. While a clear association between this polymorphism and BMI or obesity is lacking, a vast number of studies performed to date have linked the Ala allele with decreased risk for type II diabetes (reviewed in [25, 56]). The few patients described with dominant negative *PPARG* mutations present with early onset and severe insulin resistance [57] and a few studies have suggested that the His477His mutation may actually be a better predictor of type II diabetes in certain ethnic populations than the Pro12Ala mutations [32, 58, 59]. Increased fracture rates are seen in patients with type II diabetes despite an overall increase in BMD [5, 60].

In contrast, patients with type I diabetics often have osteopenia even after long periods of good metabolic control. These patients frequently have a decrease in markers of bone formation, such as serum alkaline phosphatase and osteocalcin, as this is thought to be indicative of insufficient bone accrual beginning at a very young age [60]. These observations of low bone formation are confirmed in an inducible mouse model of type I diabetes. Type I diabetic male mice have been shown to have lower BFR, and the maturation of osteoblasts from these mice is inhibited [61]. PPARG expression is shown to be increased in concert with an increase in marrow adiposity in these same mice, as well as other markers of adipocyte maturation, suggesting a mechanism for the low bone mass seen in type I diabetes [61].

Leptin (gene symbol *Lep*), a hormone secreted by adipose tissue, is thought to inhibit bone formation, as evidenced by the fact that both the *ob/ob* (leptin-deficient) and *db/db* (leptin-receptor-deficient) mice have increased bone mass and increased bone formation rate [62]. It is thought that leptin mediates its actions on bone via the sympathetic nervous system [63]. It has been proposed that PPARG suppresses *Lep* gene expression, as expression of *Lep* is increased in the *Pparg* ^+/−^ mice [64], providing yet another mechanism by which PPARG may influence the biology of bone. In humans, the His477His polymorphism has been shown to be associated with plasma leptin levels in obese subjects, yet it may be argued that this is more a reflection of the effects of PPARG on adipose tissue mass [30].

## SUMMARY

PPARG is indisputably important for bone acquisition as is clearly demonstrated by the phenotype of the *Pparg* ^+/−^ mouse. While a promising start has been made with regard to the usefulness of genetic typing for *PPARG* as predictor of BMD and fracture risk, too few studies have been completed for any conclusive statements to be made. The associations between *PPARG* and three major influences on BMD, leptin, obesity, and diabetes, are encouraging. Genetic mouse models of low BMD, such as SAMP6 and 6T, are invaluable tools for the further study of PPARG in bone.

## Figures and Tables

**Table 1:** A summary of the SNP alleles and associated bone phenotypes as studied to date in humans. The alleles are given in parenthesis after the SNP number with the major allele in the study population listed first. For SNP rs2960422 (∗) no allele frequency in this population was reported by the authors. SNPs rs11512999, rs709150, and rs1175544 (∗∗) showed no association with either BMD or BUA when analyzed separately, but an association with BMD was found for the haplotype of rs11512999 (A), rs709150 (C), and rs1175544 (C) in women.

SNP	Allele	Study population	Phenotype	Reference

His477His	C/T or T/T	Postmenopausal	Increased total	[26]
(rs3856806, C > T)		Japanese women	body BMD	
His477His	any	Pre-and Postmenopausal	No association	[27]
(rs3856806, C > T)		Korean women	with BMD	
rs2960422∗	A/G	Men and women in	Increased risk for low	[28]
		mainland China	BMD in premenopausal women only	
rs1805192 (C > G)	C/C	Caucasian men and women	Site-specific higher BMD in	[29]
			females and lower in males	
rs4684848 (G > A)	any	Caucasian men and women	No association with BMD	[29]
rs1151999 (A > C),	A, C, and C	Caucasian men and women	Site-specific	[29]
rs709150 (C > G) and	alleles inherited		lower BMD in	
rs1175544 (C > T) ∗∗	as a block only		women	
rs1152004 (T > C)	any	Caucasian men and women	No association with BMD	[29]
rs1175381 (T > C)	T/C or C/C	Caucasian men and women	Site-specific lower BMD in women	[29]
rs1186464 (A > G)	any	Caucasian men and women	No association with BMD	[29]
